# Incidence of Peri-Implantitis, Technical and Biological Complications of Single Implants Placed with Flap or Flapless Surgery—A 10–12-Year Case-Series

**DOI:** 10.3390/jcm12113668

**Published:** 2023-05-25

**Authors:** Emitis Natali Naeini, Mandana Atashkadeh, Wolfgang Jacquet, Jan D’Haese, Hugo De Bruyn

**Affiliations:** 1 Department of Periodontology and Implantology, Department of Dentistry, Radboud University Medical Centre, 6525 GA Nijmegen, The Netherlands; 2 All Saints Green Dental Practice, Norwich NR1 3LY, UK; 3 Department of Surgical Clinical Sciences CHIR-ORHE, Faculty of Medicine and Pharmacy, Vrije Universiteit Brussel, 1050 Brussels, Belgium; 4 Department of Educational Sciences EDWE-LOCI, Faculty of Psychology and Educational Sciences, Vrije Universiteit Brussel, 1050 Brussels, Belgium; 5 Department of Periodontology and Oral Implantology, Faculty of Medicine and Health Sciences, University of Ghent, 9000 Gent, Belgium

**Keywords:** flapless surgery, long-term, peri-implantitis, single implant, TiUnite surface, technical complications, biological complications

## Abstract

Background: Long-term follow-up of single implants and crowns is scarce, especially when inserted using flapless surgery. Aim: Evaluate survival, peri-implantitis incidence, and technical/biologic complications of solitary implants/crowns after 10–12 years of function. Material and methods: 49 patients with 53 single implants, initially operated with a one-stage flap (F) or flapless (FL) surgery and delayed loading, were recalled. Implant survival, radiographic bone-level changes compared to baseline, peri-implant health, and soft tissue aesthetics were registered. Differences in implant level between and within groups were statistically tested using the Mann–Whitney U-Test and Wilcoxon Signed Ranks Test, respectively. Results: 36 patients with 40 implants were reassessed, yielding 100% implant and 97.5% crown survival. The bone loss in F (*n* = 19) was 0.56 mm (SD 0.89; range −0.9–2.02) and −0.85 mm (SD 0.98; range −2.84–0.53) in FL (*n* = 21), indicative of bone gain in FL (*p* = 0.003), the latter due to a difference at baseline but bone-level was comparable (*p* = 0.126). Groups were comparable for probing pocket depth (PPD); (3.32 vs. 3.19 mm), Bleeding Index (BI); (0.15 vs. 0.22), and gingival recession; (0.38 vs. 0.17 mm). According to international criteria, the peri-implantitis incidence was 0%, but 32.5% of the implants/crowns experienced biological or technical complications irrespective of surgical technique. Conclusions: Solitary implants and crowns show good long-term clinical outcomes and peri-implant health. Flapless surgery is a good alternative to conventional in straightforward cases with sufficient bone volume and proper treatment planning.

## 1. Introduction

Over the last two decades, oral implantology has progressed, resulting in a simplification of the surgical procedure and a reduction in the treatment period. Comparable results have been achieved in the switch from two-stage surgery to one-stage [1,2,3,4]; moderately rough and bioactive implant surfaces [5] have facilitated early- [6,7,8] and immediate loading [9]. Immediate implant placement became possible in properly selected patients [10]. In addition, minimally invasive interventions by means of flapless surgery gained popularity.

Regarding the survival of single-tooth implants, systematic reviews reported a 5-year survival of 96.8% [11] and a 10-year survival of 95.2% [12]. The incidence of peri-implantitis was found in 9.7% after 5 years [11]. In a prospective follow-up of moderately rough single implants placed in a healed ridge, a 100% survival rate was reported after 8–10 years [13]. Another clinical study on moderately rough surfaces yielded 98.5% survival in the maxilla and 97.2% in the mandible after 10 years. No implants failed after 10 years of follow-up [14]. Others reported 91.5% survival after 16–22 years [15] and 96.8% after 18 years [16]. It can be postulated that single implant treatment is a predictable long-term solution.

Generally speaking, the incidence of biological and technical complications of single implants is underreported in the literature [13], especially in long-term data. A systematic review reported 0.14% implant fractures, 12.7% screw or abutment loosening, 0.35% screw or abutment fracture and 4.5% ceramic or veneer fractures after 5 years [11]. Another systematic review with meta-analysis [12] calculated 7.1% soft tissue complications after 5 years, with 5.2% of the implants showing bone loss above 2 mm. Technical complications occurred in 8.8% of the restorations, with screw-loosening (4.1%) and fracture of the veneering material (3.5%). The 5-year aesthetic complication rate amounted to 7.1% [12]. Raes et al. reported that after 8–10 years, 10% of patients with biological complications (such as fistula, signs of progressive bone loss, or pain), and 31% with one or more technical complications, including loosening of abutment screws, crown loosening, chipping, or crown fractures [13]. It is unsurprising that over time the complications increase, with 66% of crowns experiencing at least one complication during 16–22 years of follow-up [17] and 16.2% crown replacement after 18 years [16]. It can be concluded that biological and, particularly, technical complications are frequent [11,12,13,16,17].

The positivity towards the simplification of surgical procedures in the last 10 years has been affected by the often alarming reports of biological complications. In particular, those caused by peri-implantitis, which is characterized by inflammation of the peri-implant mucosa and the progressive loss of supporting bone [18,19]. It should be noted that case definitions for peri-implantitis vary substantially, which complicates the comparison between studies [20]. A recent consensus report on the Workshop on the Classification of Periodontal and Peri-Implant Diseases and Conditions [21] stated that a diagnosis of peri-implantitis requires the presence of bleeding and/or suppuration on gentle probing, increased probing depth compared to previous examinations, and the presence of crestal bone loss beyond initial bone remodeling. If data from previous examinations are lacking, then the diagnosis can be based on a combination of the presence of bleeding and/or suppuration on gentle probing, probing depths of ≥6 mm, and bone levels ≥3 mm apical to the most coronal portion of the intraosseous part of the implant [21]. A critical review reported that the overall prevalence of peri-implantitis, after a mean follow-up time of 5 years, was between 0% and 39.7%, based on 15 different case definitions [22]. For single implants, peri-implantitis prevalence is reported to be 9.7% after 5 years [11], 6.9% after 8–10 years [13], and 5% [15] after 16–22 years.

The benefits of flapless implantation, in comparison with conventional surgery, have been well reported in the literature and include less traumatic surgery resulting in less post-operative bleeding, reduced swelling of the mucosa, consequently less pain and rapid postsurgical healing [23,24,25], less time consuming [26], a minimized risk of changes to the marginal bone level due to avoiding raising a flap, and the facilitation of soft tissue management during or after implant surgery [27]. The main disadvantage of flapless implantation is that it is not possible to properly assess the volume or topography of the underlying bone, thus increasing the risk of perforations which could result in major aesthetic problems and may even lead to the loss of the implant [28]. A study by Van de Velde et al. [29] investigated the risk of perforation in a pre-clinical model in which specialists, dentists and students were asked to carry out dental procedures to insert flapless implants in a ‘free-handed’ manner. In 59.7% of cases, perforation of the bone was confirmed. The advent of cone-beam computed tomography has allowed developments in digital implant dentistry and increasingly sophisticated surgical stents to be created, which have been recommended for the flapless surgical approach in an effort to achieve ideal implant positioning [30]. Another concern regarding flapless implantation is the risk of forcing soft tissue into the osteotomy during preparation [31]. This could inhibit the osseointegration of the implant. Healing of the peri-implant tissue in flapless implantation has been studied in experimental animal studies at a histological level [32], and it was concluded that no soft tissue was forced into the osteotomy, nor was the osseointegration of the implant endangered. Additionally, there is also a potential for thermal damage as a result of reduced access for irrigation during the osteotomy preparation [25,33].

An up to 17-year retrospective analysis of a minimally invasive, flapless approach analyzed 18,945 implants in 7783 patients [34]. Cumulative survival rates after 1, 3, 5, and 10 years were 98.5%, 97.7%, 96.7%, and 93.0%, respectively. 92% of the implants analyzed were placed using a minimally invasive flapless approach. Survival rates were comparable for flapless and conventional surgery. A Cochrane systematic review [35] concluded that flapless surgery could be as successful as conventional surgery, assuming that the procedure was carried out by an experienced clinician on very carefully selected cases. However, studies with longer follow-up times that also report on peri-implantitis are needed in order to compare the success and survival of implants placed conventionally or flaplessly in the long term.

## 2. Aim

This clinical study aimed to evaluate the survival, marginal bone level changes and incidence of peri-implantitis around solitary, moderately rough implants inserted using flapless or conventional surgery. A further objective is to assess technical and biological complications.

## 3. Material and Methods

### 3.1. Study Group

In total, 53 solitary Nobel Biocare^®^ TiUnite™ implants were inserted in 49 patients in a private dental practice in Norwich, England. Details of the original study group, surgical procedure and post-operative protocol have been described in detail previously [28,36]. In brief, the cases were non-randomized, consecutively treated with conventional mucoperiosteal flap (F) or flapless surgery (FL), the latter by perforating the soft tissue with a pilot drill to create a punch. Smokers were not excluded. Three-dimensional radiographs were, at that time, not available; hence the surgeon (HDB) decided, based on a subjective analysis of bone volume, to place the implant flaplessly or conventionally with a mucoperiosteal flap. Healing abutments were placed immediately in conjunction with implant surgery. Final screw-retained or cemented crowns were placed within 6 months.

The principal dentist (MA) contacted the study group for re-evaluation of the implants after 10–12 years of function. Thirty-six of the original patients took part in the follow-up research. One independent researcher (ENN) carried out the collection of data, as well as the clinical inspection. This research protocol was run in agreement with the Declaration of Helsinki [37]. The Ethical Committee of Norfolk and Suffolk primary and community care research office regarded the research as a service evaluation. Thirty-six out of the original 49 patients took part in the follow-up research, all of which signed an informed consent form for participating and agreed that their personal data were used for a scientific report, the latter according to the Ghent University Hospital’s ethical committee requirements.

### 3.2. Clinical and Radiographic Evaluation

The following variables were recorded: patient details including an updated medical history, implant details, radiographs of the implant using the long-cone parallel technique, Silness–Loë plaque and bleeding indexes [38], a 6-point pocket chart, gingival recession, a soft-tissue evaluation based on the papilla index [39], and the gingival biotype [40]. Complications were subdivided into technical, biological, and aesthetic complications.

Baseline peri-apical radiographs had been taken on average 4.5 months after the operation. In most cases, this was to check the correct insertion of the healing abutment or impression coping. All radiographs taken after the crown insertion and during the regular check-ups were also evaluated. Seeing as there was no fixed interval for radiographs to be taken of the implants, the radiographs available were divided into intervals of 0–6 months, 7–12 months, 2–4 years, 6–9 years, and 10–12 years after implant surgery. Radiographic analysis was carried out by a clinician not involved in the surgical interventions. The software used for this was AxioVision Rel. 4.8 (Carl Zeiss MicroImaging GmbH, Jena, Germany), using an accuracy of 0.01 mm. The known distance between the implant thread (0.6 mm) was used for the calibration of the radiographs. The implant-abutment connection was determined as the baseline reference point (0 mm), from which point the closest bone-implant contact was measured.

Due to mesial and distal bone levels being statistically comparable, the mean of both was used as the implant bone level value. Thus, the bone loss after 10–12 years was calculated by deducting the bone level measured on the baseline radiographs from that measured on the radiographs during the 10–12-year interval. If the bone loss was negative, this indicated bone gain or the regrowth of bone towards the reference point.

Peri-implantitis was diagnosed according to the combined diagnosis of the bone level of 3 mm apical to the reference point, as well as a probing depth above 5 mm combined with bleeding on probing [21].

### 3.3. Statistical Analysis

Comparisons between F and FL were statistically tested using the Mann–Whitney U-Test, and changes over time within the groups using Wilcoxon Signed Ranks Test. The statistical analysis was carried out using SPSS (version 24.0). Although only two patients had received multiple implants, the number of implants (*n* = 40) was used as the unit for the statistical analysis.

The correlation between the bleeding and plaque index and the bone level around the implants was tested by means of the Spearman Correlation Test. 

The Wilcoxon Signed Ranks Test was used to test the difference between the mesiodistal average of the papilla height and the difference between the total average of gingival recessions for the flap and flapless groups.

## 4. Results

### 4.1. Study Group

**Scheme 1:**
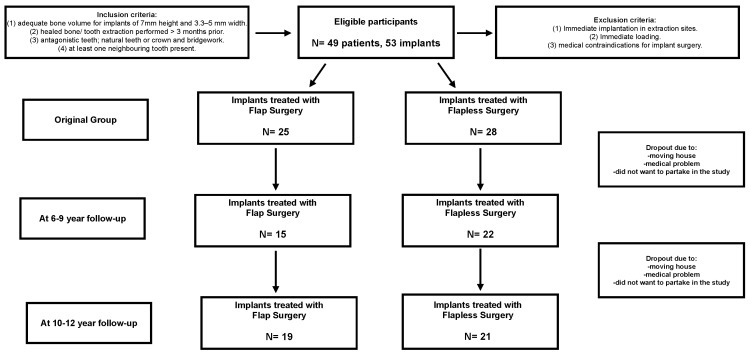
Scheme showing the flow of patients and implants treated using the Flap or Flapless treatment protocol from the time of placement until the current follow-up, including the number of drop-outs per group at the time of recall.

Thirty-six patients participated in the current follow-up study, of which 16 were women, and 20 were men. Of the 13 patients who failed to attend, six had moved house in the interim, two patients were no longer attending the clinic due to medical problems, and five patients were not prepared to be a part of the study, although their implants had survived. Scheme 1 summarizes the flow of patients and respective treatment protocol, from the time of placement, until the current follow-up. More patients from the Flap group were seen during the current follow-up than at 6–9 years, either due to agreeing to participate this time or due to their contact details being updated since the last interval.

The average age of the evaluated patients was 64.5 years (SD 13.2; range 31–89). Due to the fact that only two patients had two implants, and one had three implants, the number of implants (*n* = 40) was used as the unit for the statistical analysis. Aside from the eight patients who are no longer attending the clinic and whereby no information is available, implant survival was 100% after 10.9 years (range 9.9–12.5).

### 4.2. Treatment Protocol

**Figure 1:**
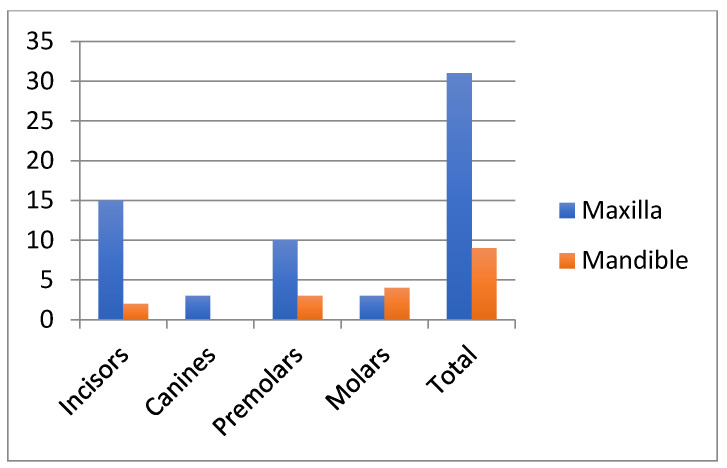
Number of implants per tooth position and jaw location.

Nineteen implants were inserted using flap surgery (F), and 21 were inserted using flapless (FL) surgery. Thirty-one implants (72.5%) were inserted in the upper jaw, and nine (27.5%) in the lower jaw. Five patients with six implants smoked at the point in time the re-evaluation of the implants was carried out. Details on implant locations are specified in Table 1 and Figure 1.

Implant specifications are given in Table 2. Thirty-three of the 40 implants (82.5%) were Brånemark external hex regular platform implants with diameters of 3.75–4.0 mm; six implants (15%) were narrow platforms with diameters of 3.3 mm; one implant (2.5%) was a wide platform with a diameter of 5 mm. Thirty-six implants (9%) were 11.5 mm or longer, as shown in Table 3.

Thirty-seven of the 40 crowns (92.5%) were metal-ceramic crowns, and 3 crowns (7.5%) were all ceramic. Thirty crowns (75%) were screwed, and 10 crowns (25%) were cemented. Eighteen crowns (45%) were not in occlusion.

### 4.3. Bone Level

**Figure 2:**
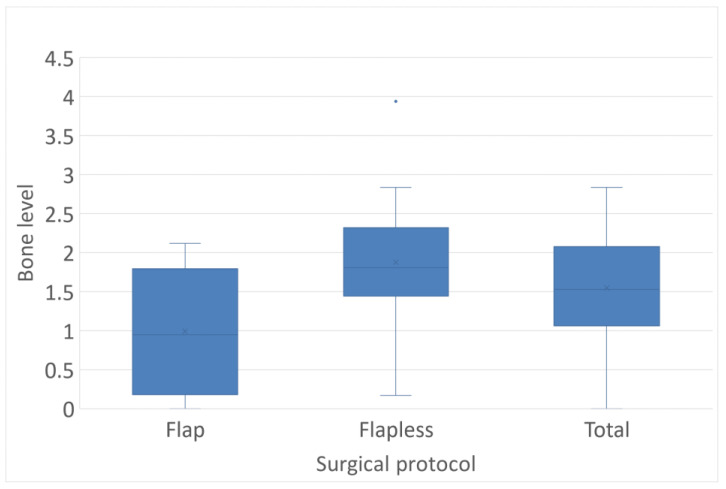
Boxplot showing 10–12 years median bone level and interquartile range in mm, as measured from the implant-abutment interface (reference point), in the flap group (*n* = 19) and flapless group (*n* = 21). There is no statistically significant difference between F and FL (*p* = 0.126).

The Baseline radiographs were taken on average 4.5 months after implant surgery (range 0–6 months). There was no statistically significant difference between F and FL with respect to the bone level at 10–12 years (*p* = 0.126). Results are summarized in Table 4. Figure 2 shows a boxplot of the bone level after 10–12 years for both treatment groups as well as the combined group.

**Figure 3:**
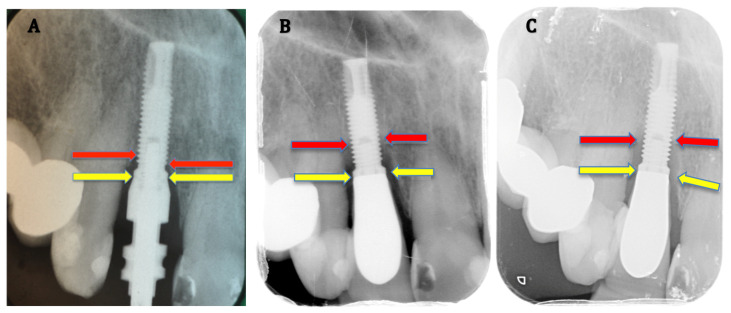
Peri-apical radiographs of an implant (corresponding to the outlier in Figure 2), which represents the worst radiographical image of bone loss in this study, at the following time points: (**A**) 6 months after implant placement, (**B**) 7 years 11 months in function (**C**) 11.5 years in function. The yellow arrow indicates the reference point from where the bone level was determined. The bone level is indicated by the red arrow.

Figure 3 shows peri-apical radiographs of implant 7, which represents the worst radiographical image of bone loss in this study, and shows the radiographic evolution from the baseline. The last 2 radiographs, although taken 3 years apart, show the bone level to be stable, which supports the statistical findings that no further changes in bone level were seen since the last published paper on this patient group [36].

**Figure 4:**
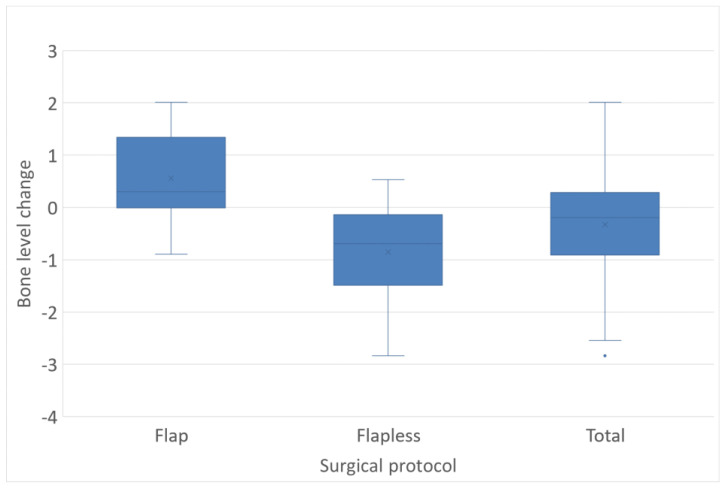
Boxplot showing the median bone level change in mm in the flap- (*n* = 19) and flapless (*n* = 21) groups, as well as for the combined group after 10–12 years. An increase in mm (above zero) suggests an increase in distance between the bone level to the implant-abutment interface, indicative of bone loss. There is a statistically significant difference between both groups (*p* = 0.003).

A statistically significant difference was found between the groups when considering bone level changes (defined as bone loss) from baseline (*p* = 0.003). Figure 4 shows a decrease in the distance from bone-to-implant level to the implant-abutment interface in the FL group, indicative of bone gain towards the implant-abutment interface. Results are summarized in Table 4.

### 4.4. Clinical Outcomes

The results show no statistically significant difference between the groups regarding probing depths, bleeding scores, gingival recession, or mesial and distal papilla heights. Therefore, these variables will be analyzed and reported regarding the combined group (Table 4).

### 4.5. Peri-Implant Health and Peri-Implantitis

**Figure 5:**
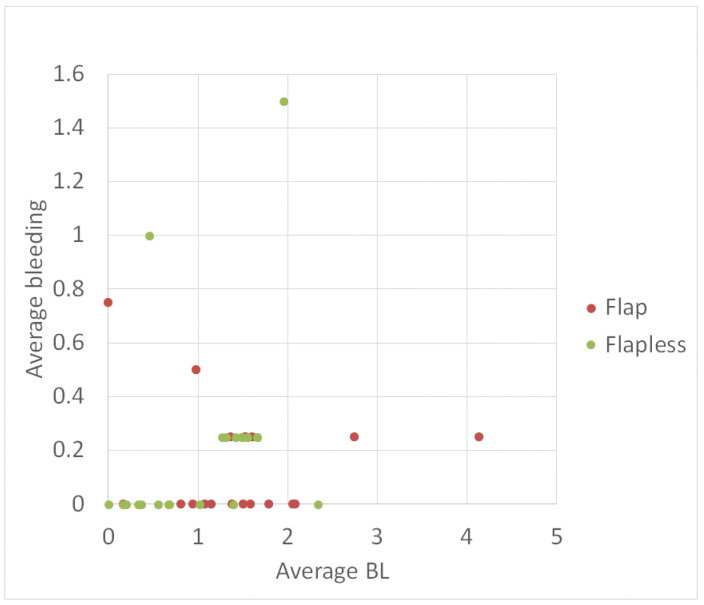
Scatter graph showing the correlation between Bleeding Index and bone loss for the whole group and surgical protocol.

When looking at the relationship between bleeding and bone loss around implants, little correlation was seen (see Figure 5).

Table 5 shows the implant distribution divided into pocket depth, with or without bleeding, and bone loss as measured from the day of loading. Twenty-six out of 32 implants (81%) exhibited a bone loss that did not exceed 1 mm, despite bleeding on probing in 13/32 implants. One implant showed an average bone loss of 4.13 mm in the F group (shown in Figure 2). Although six implants (18.8%) had lost bone beyond 1 mm, they did not have pockets above 5 mm. Furthermore, they displayed no bleeding on probing. Based on the cross-table combining bone loss and pocket formation, it can be deduced that no implants exhibited peri-implantitis according to the 2017 consensus report 21, considering there were no implants with a bone level of 3 mm apical to the most coronal portion of the intraosseous part of the implant, as well as a probing depth of >5 mm combined with bleeding on probing 21. However, mucositis was present in 40.6% of the 32 implants. Eight radiographs were not readable; therefore, bone loss could be measured in 32/40 implants.

### 4.6. Complications

Twenty-seven out of 40 implants (67.5%) showed no complications since the implant was inserted. Of the 13 implants with complications, five of them experienced a technical complication (crown fracture, cement loosening, infra-position, and chipped porcelain), seven experienced a biological complication, and seven experienced aesthetic problems (recession, wear of the porcelain). Seven implants had one complication, five implants experienced more than two complications since placement, and one implant had five complications since placement. 11 of the 13 complications were followed up without any intervention, 1 implant underwent crown removal and replacement (implant 29), and one was treated with non-surgical peri-implant therapy (implant 7). The first complication occurred on average 2.7 years after insertion of the implant. During the entire follow-up period, only one crown was replaced. There was no correlation between bone loss, probing pocket depth, and the type of restoration. The complete results are summarized in Table 6. The number of complications did not differ significantly between the F and FL groups (*p* = 0.737).

## 5. Discussions

This study is one of the few studies that has compared flapless implant surgery with conventional surgery whilst utilizing the same loading protocol for the implants in both groups. The aim of this study was the long-term evaluation of the clinical outcome of solitary implants inserted using flapless or conventional (flap) surgery, namely the marginal bone level, implant survival rate, peri-implant health, and complications. Thirty-six patients of the original study group consisting of 49 patients participated in the follow-up research 10–12 years after the insertion of the implants. The drop-out percentage was 26.5% at the patient level and 24.5% at the implant level. This is a relatively low drop-out percentage considering that this was a long-term retrospective study and is inevitable with time, even with the best study design and conduct [41]. As the outcome is not dependent on the extent to which a patient did or did not participate in the follow-up study, it is possible to postulate that this group of patients is representative of the original group [41]. Furthermore, the details relating to the survival of the implants were obtained from all patients when they were contacted by phone, despite being unable or unwilling to participate in the clinical research.

This study recounts a 100% survival rate of solitary implants without stringent exclusion criteria, such as the exclusion of smokers or patients with a history of periodontitis. This result is comparable to several other studies [26,42,43,44,45] that demonstrate that flapless implantation is a reliable treatment method with survival percentages of 97–100%.

The significant difference in bone level change between groups can be attributed to excessive countersinking in the flapless group [28]. By doing this, too much bone can be removed, thus causing the coronal part of the implant not to be in close contact with the bone. This may possibly result in under-scoring the bone level on the baseline radiographs. Bone remodeling allows a gain in bone in the flapless group and a consequent reduction in the distance between the implant-abutment connection and the first bone-implant contact in order to re-establish the biological width. It is probable that in the flapless group, the excessive countersinking was carried out as a consequence of the lack of direct sight of the osteotomy. Hence, part of the transmucosal (supracrestal) section of the implant is slightly below the crestal bone level. In this case, bone remodeling around the neck of the implant is normal [46]. The visual inspection of the bone and the insertion of the healing abutment was not compromised in the flap group, so the healing abutment could be inserted without excessive countersinking.

Results were comparable between both treatment groups with regards to probing depth and bleeding index, and as such, the patient group was reported on as a whole. One may expect to see a correlation between the pocket depth and the marginal bone level; however, this was not the case. This implies a limited diagnostic value of probing for the detection of ongoing bone loss, as long-term human studies have shown that the probing depth of healthy peri-implant mucosa can, in fact, reach up to 6 mm [16,47,48]. Stable probing depths in the long term may perhaps have greater diagnostic value, implying an absence of bone loss and, thus, a stable condition in the peri-implant environment. The evaluation of the bone loss over a certain time period provides a better picture of the evolution of the marginal bone level, the occurrence of ‘ongoing bone loss’, and, thus, peri-implantitis. A recent consensus report on the Workshop on the Classification of Periodontal and Peri-Implant Diseases and Conditions [21] stated that a diagnosis of peri-implantitis requires the presence of bleeding and/or suppuration on gentle probing, increased probing depth compared to previous examinations, and the presence of bone loss beyond initial bone remodeling. If data from previous examinations are lacking, then the diagnosis can be based on a combination of the presence of bleeding and/or suppuration on gentle probing, probing depths of ≥6 mm, and bone levels ≥3 mm apical of the most coronal portion of the intraosseous part of the implant [21].

If we define peri-implantitis as bone loss > 2 mm [49] in combination with a pocket depth > 5 mm [50] as well as bleeding after probing, then not a single implant in this study group showed signs of peri-implantitis. As mentioned in the results, one implant (#7) experienced more bone loss than others and was registered as a biological complication, whereby it was successfully treated with non-surgical peri-implant therapy. The bone loss around this implant was, therefore, no longer ongoing.

According to the aforementioned criteria, the moment of loading of the implant is defined as the baseline [21]. In this study, the baseline radiographs were taken on average 4.5 months (range 0–6 months) after the implant surgery, some radiographs being taken just after the insertion whilst others were taken after the physiological bone remodeling. Seeing as the initial bone remodeling occurred 3–6 months after implant insertion [51], this means that for some implants, the changes determined in marginal bone level may possibly have been a part of the initial physiological bone remodeling after implant surgery. It should be noted that this effect is most likely to be present in both groups and so does not affect their comparison. However, since the healing abutment was placed at the time of implantation, initial bone remodeling started at the time of placement, so a baseline at 6 months can be used to measure functional/ongoing bone loss [52]. It is important to differentiate between the initial physiological bone remodeling that occurs up to 3–6 months after the implantation and ‘ongoing bone loss’ that is defined by Albrektsson and Isidor (1994) as the continuous bone loss around a functional implant after osseointegration has taken place and is therefore alarming. The assumption is that bone loss that occurs after the initial bone remodeling is the consequence of bacterial infection [53]. Ramanauskaite et al. [50] confirmed that a pocket above 6 mm in combination with bleeding after probing is an indicator of a greater risk of peri-implant pathology. This is due to the biological width around the implant being 4 ± 1 mm, so a pocket depth above 6 mm no longer falls within the margin of the biological width. However, this should be supported by radiographic analysis.

Furthermore, the Spearman Correlation Test showed no correlation between the bleeding index and bone loss in this study. Bleeding after probing is a sign of mucositis that may or may not be accompanied by bone loss [47,54]. Interestingly, in an 18-year follow-up study, Dierens et al. [15] demonstrated that bleeding on probing is a bad predictor for bone loss or peri-implantitis. Additionally, in a recent paper by Coli et al. [48], it was concluded that there is inadequate evidence to recommend bleeding on probing as a useful clinical variable for assessing or predicting attachment loss. The results from this study support that. That being said, it does not mean that bleeding of the peri-implant gums should be neglected. It has been demonstrated that mucositis is a predictor for future bone loss [55]; therefore, patients should be motivated to maintain high levels of oral hygiene and attend regular recall appointments to re-evaluate the peri-implant tissues.

Over the last 10 years, more and more interest has been shown in other treatment outcomes, which represent the modern demands of our society [56]. The aesthetic outcome has become a key concern in modern dentistry, and many objective parameters have been used to assess this outcome; gingival recession is one of said parameters [56,57]. The marginal gingival level tends to mimic the bone level underneath (biological width). One may expect that the conventional flap surgery may cause more marginal bone loss due to impeding the supraperiosteal blood supply as a result of raising a mucoperiosteal flap. Studies have established that flap reflection tends to produce bone resorption around natural teeth [58]. This is particularly interesting when considering the long-term outcomes of implant surgery as it affects the aesthetics of the implant-retained prosthesis in the long run. The results in this study showed no statistically significant difference between groups (*p* > 0.05) and corresponded to results found in various studies [59]. Long-term studies reporting on these outcomes are limited, and the majority focus on immediate implant placement (Type I) [40,60] rather than Type IV placement, as reported in this study.

It is important to consider the survival rate of implant-supported single crowns when choosing between various treatment options for replacing a single tooth [12]. One meta-analysis reveals a survival rate of 89.4% after 10 years for implant-supported single crowns [12]. In the present study, one crown was replaced, bringing survival to 97.5%. Another meta-analysis revealed the 10-year survival of conventional fixed prostheses is 89.1% [61] and 81.8% for cantilever fixed prostheses [62], thus rendering a single crown supported by an implant a recommended treatment modality for single tooth gaps.

Clinicians must be aware that complications do occur to various extents. The literature reports soft tissue and aesthetic complications very inconsistently and without any standardization and classification [12]. A meta-analysis yielded a 7.1% cumulative biological complication rate after 5 years [12]; however, it should be mentioned that this figure is lower than the 9.7% reported in previous systematic reviews [11], showing a difference when newer studies are analyzed. These figures are lower than the 17.5% biological complication rate in our study, possibly due to the fact that the follow-up time is more than double compared to the aforementioned studies. These complications have to be considered and strengthen the need for a well-established maintenance program [12]. In terms of technical complications, the results of the present study are comparable to those found in the literature [11,12,63], with the most common technical complications being abutment or screw-loosening [12]. As mentioned previously, aesthetic outcomes and complications are a major focus from both the patient and clinicians’ perspective; however, there is a lack of standardized parameters and indices to evaluate the aesthetic appearance, and a large heterogeneity exists between the different studies, limiting the scientific value of the calculated aesthetic complication rate as it is often based on various measurements and parameters [12]. The 5-year cumulative aesthetic complication rate reported in a meta-analysis is 7.1% [12]. Unsurprisingly, this figure is lower than the aesthetic complication rate reported after 10–12 years in the present study. A scientific consensus on an accepted and reproducible method to evaluate the aesthetic outcome of single crowns on the soft tissue level and on the level of the crown itself is needed [12], preferably with longer follow-up times.

A systematic review concluded that cement-retained constructions showed more serious biological complications (implant loss, bone loss > 2 mm) as compared to screw-retained constructions, which showed more technical problems [63]. A meta-analysis found that after 10 years, the type of fixation of the reconstruction did not have any significant influence on the estimated rate of biological complications [12]. A more recent systematic review had similar findings; when considering “all fixed prostheses,” single crowns, and fixed partial prostheses, a higher rate of technical and biological complications was found for cement-retained prostheses [64]. The incidence of technical complications was more dependent on prosthesis and retention type rather than abutment material [64]. It can be established that screw-retained reconstructions are more easily retrievable than cemented reconstructions, thus making technical and, eventually, biological complications more easily treatable. Additionally, implants with cement remnants in periodontitis-susceptible patients may be more likely to develop peri-implantitis [65]. For this reason, screw-retained constructions are preferable [63,64,65].

The overall number of complications, combined as aesthetic, technical or biological ones, was 32.5% after 10–12 years of function. This is an increase compared to the 6–9-year interval, reported as being 24% [36]. However, the consequence was not as significant for the patient. The clinicians were able to treat the complication either with surgical intervention (1/13) or crown replacement (1/13). In the rest of the cases experiencing a complication, an intervention was not deemed necessary (11/13). Therefore, one could suggest that solitary tooth replacement by means of an implant is a reliable treatment method that has limited problems associated with it, both for the patient and the clinician. When carrying out flapless implant surgery, one can expect just as few/many complications can as with conventional surgery.

### Limitations

A drawback of the current study was that it was not a randomized study with a control group but was drawn up as a retrospective one. Patients were given flapless implant surgery in cases where conditions were favorable after clinical and radiographic examinations of the available bone. It was the clinician who decided what the right choice was, depending on their surgical skill and clinical experience. In some cases, flapless surgery was planned, but in dubious cases, flap elevation was carried out. It has already been shown that the outcome of flapless implants depends heavily on the experience of the clinician and good patient selection [42]. The possible selection bias must be borne in mind when interpreting the results; however, this approach actually mirrors the decision process that would take place in daily practice. A randomised study with a control group would not be able to do this. This also explains the number of confounding factors in this study; smokers vs. non-smokers, cement-retained vs. screw-retained, as these were the patients who were in need of implant treatment at the time. The number of cases is too limited to make a distinction between these risk factors or differences in prosthetic protocols.

Another limitation of this study, despite the long follow-up period, is the small sample size. Many of the outcome variables showed significant differences; however, they did not prove to be statistically significant. The relevant question is whether the lack of a difference between the flapless and the open flap surgical procedures is a real finding or is due to the lack of statistical power, given the small number of patients per group. A meta-analysis by Chrcanovic et al. [26] also recognized this shortcoming in various independent studies [25,43,66,67,68,69].

## 6. Conclusions

The outcomes of this study demonstrate that solitary implants have an excellent prognosis with stable bone levels irrespective of the surgical technique applied after 10–12 years and that ‘free-handed’ surgery of solitary implants with adjacent teeth, which indicate the direction of surgery, are good alternatives to the more expensive and more complex guided surgery. Flapless surgery must be preceded by stringent patient selection based on the quantity of available bone, good pre-surgical planning and clinical experience.

This treatment modality for the restoration of a single tooth gap can be deemed to be a safe and reliable option, despite the varying rates of technical, biological, and aesthetic complications, which are to be expected.

## Figures and Tables

**Table 1:** Number of implants per tooth type and jaw location.

Implant Position	Maxilla	Mandible	Total
Incisors	15	2	17
Canines	3	0	3
Premolars	10	3	13
Molars	3	4	7
Total	31	9	40

**Table 2:** Implant type divided per treatment group.

Implant Platform (mm)	Flap	Flapless	Total
NP (3.3)	3	3	6
RP (3.75–4.0)	14	19	33
WP (5)	0	1	1
Total	16	22	40

**Table 3:** Implant length divided per treatment group.

Implant Length (mm)	Flap	Flapless	Total
10	1	2	3
11.5	1	3	4
13	4	8	12
15	15	5	20
18	0	1	1
Total	21	19	40

**Table 4:** Table showing descriptive results of the clinical outcomes at the time of follow-up.

		Total Patient Group	Flap	Flapless
**Number of implants**	**Follow-up**	40	19	21
**Bone level at follow-up (mm)**	**Mean** **(SD; range)**	−1.26 (0.8; −4.1–0)	−1.49 (0.9; −4.1–0)	−1.04 (0.66; −2.3–0)
**Bone loss (mm)**	**Mean** **(SD; range)**	−0.33 (1.16; −2.84–2.02)	0.56 (0.89; −0.9–2.02)	−0.85 (0.98; −2.84–0.53)
**PPD (mm)**	**Mean** **(SD; range)**	3.26 (0.87; 1.83–5.33)	3.32 (0.84; 2.17–4.83)	3.19 (0.91; 1.83–5.33)
**BOP**	**Mean** **(SD; range)**	0.19 (0.29; 0–1.5)	0.15 (0.21; 0–0.75)	0.22 (0.38; 0–1.5)
**Gingival recession (mm)**	**Mean** **(SD; range)**	0.28 (0.52; 0–2)	0.38 (0.60; 0–2)	0.17 (0.45; 0–2)
**Mesial Papilla Height (mm)**	**Mean** **(SD; range)**	3.67 (1.58; 0–8)	3.58 (1.68; 0–8)	3.76 (1.48; 0–6)
**Distal Papilla Height (mm)**	**Mean** **(SD; range)**	2.69 (1.60; 0–6)	3.05 (1.51; 0–6)	2.33 (1.68; 0–5)

**Table 5:** Number of implants with corresponding bone loss (mm) and probing pocket depth (mm). Eight radiographs were not readable; therefore, bone loss could be measured in 32 of 40 implants. The number in brackets represents implants with bleeding on probing, followed by the percentage of implants which showed bleeding out of the total number of implants (*n* = 32). None of the implants were classified as having peri-implantitis, as denoted by the dark blue square.

Bone Loss/Pocket Depth	≤3 mm	>3 mm & ≤4 mm	>4 mm & ≤5 mm	>5 mm	Total
<0 mm (bone gain)	4 (2; 6.3%)	7 (2; 6.3%)	4 (3; 9.4%)	1 (1; 3.1%)	16 (8; 25%)
≤1 mm	7 (3; 9.4%)	2 (1; 3.1%)	1 (1; 3.1%)		10 (5; 15.6%)
>1 ≤ 2 mm	2 (0; 0%)	1 (0; 0%)			3 (0; 0%)
>2 ≤ 3 mm	2 (0; 0%)				2 (0; 0%)
>3 mm		1 (0; 0%)			1 (0; 0%)
Total	15	11	5	1	32 (13; 40.6%)

**Table 6:** Table showing the surgical protocol (F = flap, FL = flapless), retention type (C = cement-retained, S = screw-retained), the total number of complications as well as complication type (technical, biologic or aesthetic), bone loss (mm) and probing pocket depth (PPD) (mm) for each implant. The table has been divided with respect to crown retention type. A negative score for bone loss indicates an increase in bone, as measured from the abutment-implant interface (NR: baseline radiograph was not readable). * refers to implant 7 (see Figure 3), which was treated successfully with non-surgical peri-implant therapy.

Implant Number	Surgical Protocol F/FL	Crown Retention	Total No. Compl.	Technical Compl.	Biological Compl.	Aesthetic Compl.	Bone Loss	PPD
2	F	C					NR	4.8
5	F	C					−0.17	2.5
8	F	C	1		1		−0.44	4.2
25	F	C	2	1		1	−1.50	3.5
14	FL	C	1			1	−0.21	5.0
19	FL	C					0.18	2.0
29	FL	C	5	4	1		2.55	2.2
35	FL	C					0.23	2.7
1	F	S					−0.05	2.2
4	F	S					−1.14	3.8
6	F	S					NR	2.7
7	F	S	2		1 *	1	−2.02	4.5
10	F	S					NR	3.2
11	F	S					NR	3.7
12	F	S					0.90	3.0
16	F	S					NR	2.5
17	F	S	3	1	1	1	−0.09	2.8
22	F	S					NR	2.3
24	F	S	3		3		NR	2.8
26	F	S					0.20	2.7
39	F	S					−2.05	4.0
40	F	S					−2.74	3.8
43	F	S					−1.28	4.7
23	FL	S	2		1	1	NR	2.8
27	FL	S					−1.95	5.3
30	FL	S	1	1			0.69	3.5
31	FL	S	1	1			0.59	2.3
32	FL	S	1			1	1.69	3.3
34	FL	S					1.28	2.7
38	FL	S	1			1	0.76	3.5
41	FL	S	1		1		0.91	3.8
44	FL	S					2.09	3.3
45	FL	S					−0.53	3.8
46	FL	S					−1.01	3.7
47	FL	S					0.11	1.8
49	FL	S					2.84	2.8
50	FL	S					1.20	2.7
51	FL	S					−0.28	3.7
52	FL	S					0.39	2.8
53	FL	S					−1.42	2.7

## Data Availability

The data presented in this study are available on request from the corresponding author. The data are not publicly available due to ethical restrictions.
